# Conceptualization and Investigation of Multicomponent Polymer Networks as Prospective Corticosteroid Carriers

**DOI:** 10.3390/gels9060470

**Published:** 2023-06-07

**Authors:** Dilyana Georgieva, Mariela Alexandrova, Sijka Ivanova, Darinka Christova, Bistra Kostova

**Affiliations:** 1 Department of Pharmaceutical Technology and Biopharmacy, Faculty of Pharmacy, Medical University of Sofia, Dunav Str. 2, 1000 Sofia, Bulgaria; dgeorgieva@pharmfac.mu-sofia.bg; 2 Institute of Polymers, Bulgarian Academy of Sciences, Akad. G. Bonchev Str., Bl. 103-A, 1113 Sofia, Bulgaria; m.alexandrova@polymer.bas.bg (M.A.); sivanova@polymer.bas.bg (S.I.)

**Keywords:** drug delivery, copolymer network, interpenetrating network, dexamethasone sodium phosphate, dermal application

## Abstract

Dexamethasone (DXM) is a highly potent and long-acting synthetic glucocorticoid with anti-inflammatory, anti-allergic, and immunosuppressive effects. However, the systemic application of DXM can cause undesirable side effects: sleep disorders, nervousness, heart rhythm disorders, heart attack, and others. In the present study, multicomponent polymer networks were developed as potential new platforms for the dermal application of dexamethasone sodium phosphate (DSP). First, a copolymer network (CPN) comprising hydrophilic segments of different chemical structures was synthesized by applying redox polymerization of dimethyl acrylamide onto poly(ethylene glycol) in the presence of poly(ethylene glycol) diacrylate (PEGDA) as a crosslinker. On this basis, an interpenetrating polymer network structure (IPN) was obtained by introducing a second network of PEGDA-crosslinked poly(N-isopropylacrylamide). Multicomponent networks obtained were characterized by FTIR, TGA, and swelling kinetics in different solvents. Both CPN and IPN showed a high swelling degree in aqueous media (up to 1800 and 1200%, respectively), reaching the equilibrium swelling within 24 h. Additionally, IPN showed temperature-responsive swelling in an aqueous solution as the equilibrium swelling degree decreased considerably with an increase in the temperature. In order to evaluate the networks’ potential as drug carriers, swelling in DSP aqueous solutions of varied concentration was investigated. It was established that the amount of encapsulated DSP could be easily controlled by the concentration of drug aqueous solution. In vitro DSP release was studied in buffer solution (BS) with pH 7.4 at 37 °C. The results obtained during DSP loading and release experiments proved the feasibility of the developed multicomponent hydrophilic polymer networks as effective platforms for potential dermal application.

## 1. Introduction

Dexamethasone (DXM) is a highly potent and long-acting synthetic glucocorticoid. It was synthesized in 1957 and is structurally analogous to other corticosteroids such as hydrocortisone and prednisolone [1]. Glucocorticoids have an anti-inflammatory, anti-allergic, and immunosuppressive effect that depends on the dose—lower doses provide an anti-inflammatory effect, while higher doses are immunosuppressive [2]. DXM belongs to Class I/III of the Biopharmaceutical Classification System (BCS) [3]. It is approved for the palliative management of leukemias and lymphomas. It impedes the function of different immune cells, so it is also used in the treatment of multiple myeloma and lung cancer, because it minimizes inflammation, reduces nausea accompanying the chemotherapy and destroys specific cancer cells [4]. Due to its anti-inflammatory and anti-allergic effects, DXM is applied in the therapy of many eye and skin diseases [5,6,7,8,9,10,11]. The mechanism of action is the activation of glucocorticoid receptors, which leads to the repression of transcription factors activated in chronic inflammation. DXM is available on the pharmaceutical market in different dosage forms, such as injections, tablets, eye drops and eye ointments, and skin lotions. The systemic application of DXM, however, can cause undesirable side effects, such as sleep disorders, nervousness, heart rhythm disorders, heart attack, and others. Good alternatives for overcoming these problems are the inclusion of DXM in nanosized drug delivery systems, and topical administration.

The majority of reported DXM-loaded nanoparticles have been prepared using its base and acetate forms, which are water-insoluble [12,13,14,15,16,17,18]. Research on the incorporation of the water-soluble dexamethasone sodium phosphate (DSP) into nanoparticles is limited. The incorporation of DSP into nanoparticles based on poly(lactic-*co*-glycolic acid), chitosan, poly(glycerol adipate), or poly(amido amines) has been found to alter the biopharmaceutical properties [19,20,21,22,23,24,25]. Our scientific group also investigated the potential of DSP-loaded nanoparticles as delivery systems. We developed cationic polyelectrolytes of different macromolecular structure using [2-(acryloyloxy)ethyl]trimethylammonium chloride (AETMAC), block or star shaped, and studied the complex formation with DSP. All copolymer structures showed high DSP loading efficiency and controlled the release process for a period of 24 h [26].

As has already been mentioned, the second alternative for overcoming the side effects of DXM is topical administration. In this aspect, hydrogels have gained a great deal of interest based on their recognized biocompatibility, flexibility, and softness, similar to natural tissue, as well as their high drug loading capacity due to the specific swelling behavior [27,28]. Hydrogels are hydrophilic polymer systems that are insoluble, but can absorb varying amounts of water depending on their properties [29,30]. Due to their three-dimensional structure and the cross-linking of polymer chains, they are physically stable [31]. Synthetic hydrogels are characterized by mechanical strength and resistance to degradation. They also have tunable properties (e.g., controlled by the degree and geometry of the cross-linking agent, or the derivatization of pendant groups) [32,33,34,35,36,37]. Hydrogels are advantageous delivery systems for hydrophilic drugs because dissolved drugs could be included in the swollen cross-linked polymer matrix [29,30,31]. A variety of network structures could be used for the development of hydrogels suitable for dermal drug delivery applications, including copolymer architectures [38,39,40] and interpenetrating (IPN) and semi-interpenetrating networks [41,42,43]. A high swelling capacity in water and in body liquids of the networks, which reduces the irritancy of the carrier, is of prime importance, along with the general requirements for biocompatibility and non-toxicity. In addition, softness and flexibility, as well as the ease of removal of the network film, are favorable, especially in the case of open wound care [44].

To our best knowledge, the majority of the literature data concerning dermal delivery of DSP is based on the use of iontophoresis [45,46,47]. There is a lack of data about the application of polymeric networks for the dermal delivery of DSP. The existing DSP-loaded polymer networks aimed at oral delivery for the possible treatment of inflammatory bowel diseases [48] or colonic delivery [49]. Therefore, the aim of the present study was the synthesis and characterization of the multicomponent polymer networks—copolymer networks (CPN) as well as IPN. In this regard, hydrophilic PDMAAM, PNIPAAM and PEO were chosen as building blocks of the networks. A synthetic approach involving redox as well as radical copolymerization in the presence of a hydrophilic macromolecular crosslinker was applied, providing CPN and IPN carriers of durable chemical structures with the potential for the dermal application of DXM.

## 2. Results and Discussion

### 2.1. Synthesis of the Multicomponent Polymer Networks

**Figure 1:**
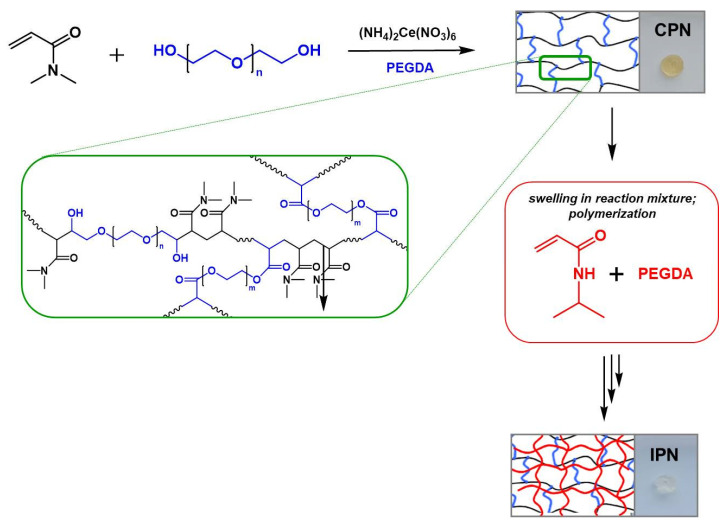
Schematic presentation of the synthesis of the copolymer network and interpenetrating polymer network.

In a previous study [50], we explored two methods for the synthesis of copolymer networks as potential transdermal drug delivery platforms: (i) the free radical polymerization of a monomer in the presence of a macromolecular crosslinker—poly(ethylene glycol) diacrylate (PEGDA); and (ii) redox polymerization of the monomer in the presence of the macromolecular crosslinker initiated by cerium(IV) ammonium nitrate (CAN) onto a hydroxyl end-caped precursor. The latter method provides the effective one-pot one-stage synthesis of complex copolymer architectures depending on the hydroxyl initiating moiety used, and has been applied in the design of a variety of block, branched, and crosslinked copolymers based on PEG, poly(vinyl alcohol) and other hydroxyl functionalized precursors [50,51,52]. In the present work, the two approaches were combined in order to develop multicomponent polymer networks for the dermal application of dexamethasone sodium phosphate (Figure 1). First, CPN comprising hydrophilic segments of different chemical natures were synthesized by applying redox polymerization of N,N-dimethyl acrylamide (DMAAM) initiated by CAN onto poly(ethylene glycol) (PEG) as a di-functional hydroxyl-containing precursor in the presence of poly(ethylene glycol) diacrylate (PEGDA) as a crosslinker. Polymerization was carried out in aqueous media at a low reaction temperature and easy network film workup. The resulting hydrophilic multicomponent network structure comprising PDMAAM and poly(ethylene oxide) (PEO) segments in the form of a film were carefully purified from reagents’ residues, and further analyzed. The soluble fraction in water calculated for CPN was 9.1% ± 0.14% and for IPN was 4.0% ± 0.20%.

In the next stage, IPN was obtained via the copolymerization of N-isopropylacrylamide (NIPAAM) with PEGDA as the crosslinker (Figure 1). A sequential IPN synthetic procedure was applied and polymerization was carried out within the pores of the first network, the reaction mixture of NIPAAM, PEGDA, and radical initiator 4,4′-azobis (4-cyanovaleric acid) being introduced in the CPN structure by swelling. Polymerization was carried out under nitrogen for 24 h, increasing the reaction temperature from 30 °C to 60 °C. The IPN thus obtained was built of two entangled multicomponent networks: one hydrophilic (CPN), and another one thermoresponsive, which comprised PNIPAAM and PEO segments and penetrated within but was not covalently bonded to CPN. Both CPN and IPN were carefully purified by prolonged swelling in deionized water, dried in vacuum until a constant weight, and comprehensively characterized.

### 2.2. Fourier Transform Infrared (FTIR) Spectroscopy

**Figure 2:**
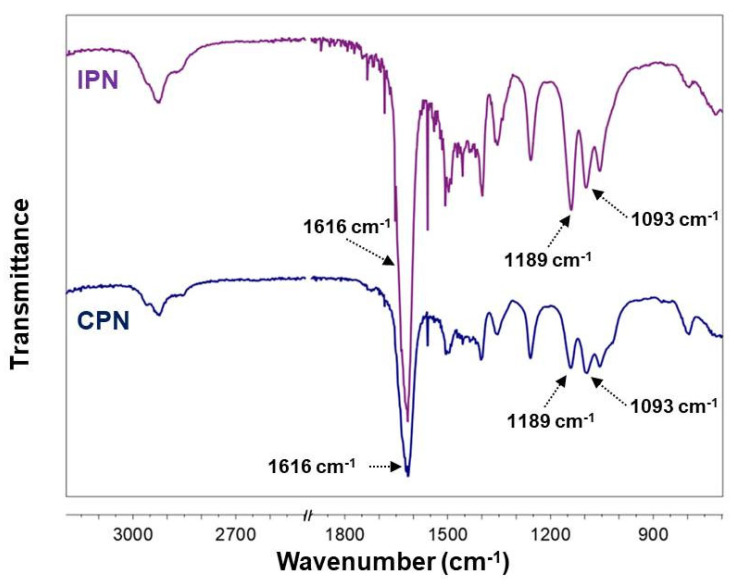
FTIR spectra of CPN and IPN.

The chemical composition of the purified CPN and IPN was evaluated by FTIR analysis. In the FTIR spectrum of the initially synthesized CPN (Figure 2), the characteristic peaks of PDMAAM and PEO segments are well visible: the strong carbonyl band at 1616 cm^−1^ was ascribed to the C=O stretching vibrations (amide I) of PDMAAM and the peak at about 1500 cm^−1^ assigned to H–C–H bending vibrations of the attached methyl groups. Strong bands at 1139 cm^−1^, 1093 cm^−1^, and 1056 cm^−1^ were attributed to the C–O–C vibrations characteristic for PEO segments. The dominant peak in the spectrum of IPN (Figure 2), also located at 1616 cm^−1^, was associated with the carbonyl stretching vibrations of amide groups in PDMAAM and PNIPAAM segments. Characteristic bands for C–O–C vibrations of PEO segments appeared in the range from 1150 cm^−1^ to 1050 cm^−1^, similar to the corresponding bands of CPN but of changed intensities.

### 2.3. Swelling Experiments

**Figure 3:**
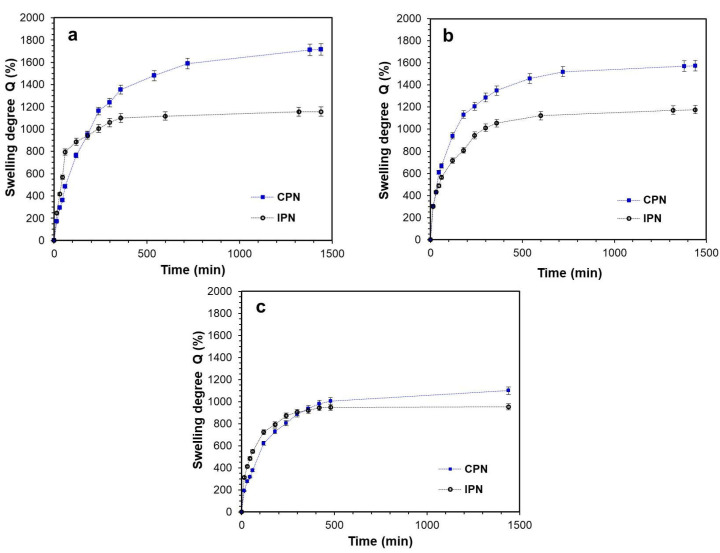
Swelling of CPN and IPN in water at 20 °C (**a**), in BS pH 7.4 at 37 °C (**b**), and in ethanol at 20 °C (**c**).

The swelling kinetics of the networks were conducted in water at 20 °C and in buffer solution (pH 7.4) at 37 °C (Figure 3). Swelling in water is investigated as a basic characteristic of any hydrophilic network structure. In this study, however, it was of particular importance because of the potential drug loading of the networks via swelling in DSP aqueous solutions. As shown in Figure 3a, the investigated platforms could absorb considerable amounts of water, taking up 1700% and 1150% for CPN and IPN, respectively. The swelling kinetics of CPN and IPN followed similar profiles, and the networks reached their equilibrium within 24 h. The observed high swelling degrees at ambient temperatures were indicative and provided conditions for controlled drug loading via a simple and convenient procedure. As expected, IPN showed lower equilibrium swelling due to the denser double network structure and lower void volume available for water molecules.

The swelling of CPN at physiological conditions (Figure 3b) followed an identical kinetic profile to those in water, although gaining a lower equilibrium swelling of 1570%. This was expected behavior considering the salt content (and ionic strength in particular) of BS, and an observed phenomenon for copolymer networks of similar nature. On the other hand, the swelling of IPN in BS at 37 °C resulted in equilibrium swelling of 1180%, almost equal to the swelling in pure water, which was in contrast to the CPN swelling behavior. It is well known that PNIPAAM gels demonstrate temperature-dependent swelling in water: PNIPAAM chains, being hydrophilic and hydrated at ambient temperatures, turn hydrophobic and collapse over the lower critical solution temperature (LCST of about 32 °C). When swelling in BS at 37 °C, it was obvious the temperature-responsive PNIPAAM segments of the second network in IPN were collapsed (hydrophobic) and provided additional volume in the IPN pore structure for the BS solution. This could explain the preserved equilibrium swelling of IPN in BS as compared to its swelling in water.

The swelling of CPN and IPN in ethanol was also conducted (Figure 3c). Ethanol was chosen as a commonly used solvent in pharmaceutical practice. It is clear that both CPN and IPN swelled equally in ethanol regardless of the copolymer composition, but much lower compared to their swelling in water and BS. This could be explained with the similar solubility of the CPN and IPN constituents in ethanol.

### 2.4. Drug Loading and DSP Content

**Figure 4:**
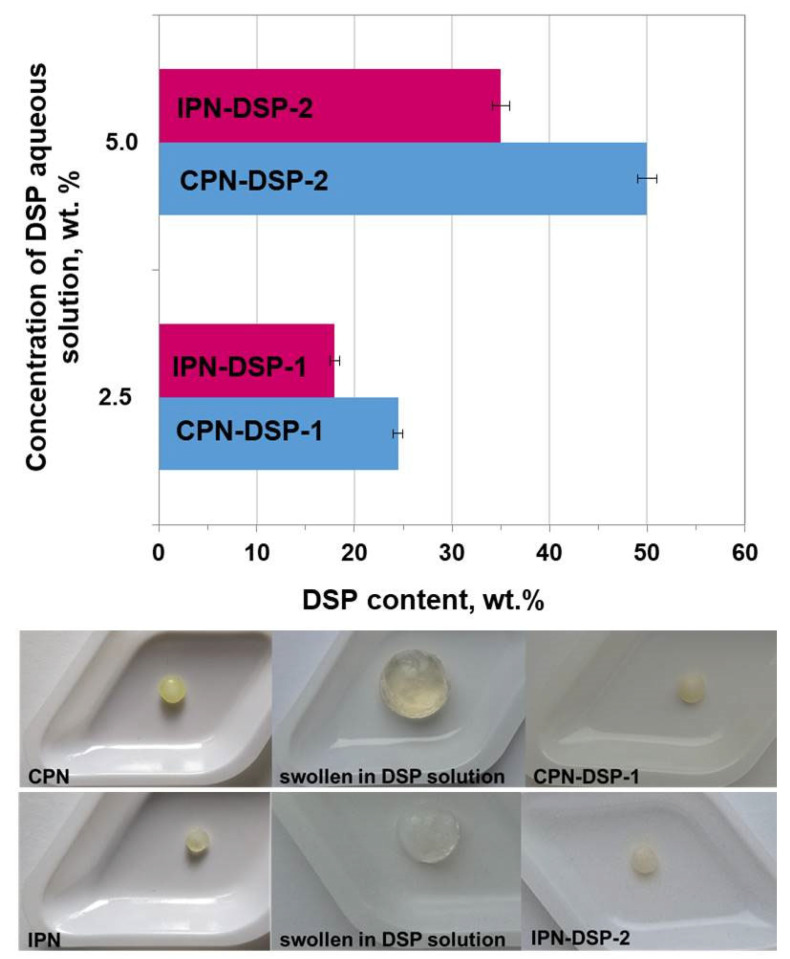
Drug loading of CPN and IPN at two different DSP concentrations, and images illustrating the drug loading process.

The results presented in Figure 4 indicate that the drug loading of the different networks varied significantly. IPN showed a lower DSP content compared to CPN. This was expected, taking into consideration the results from the swelling studies. As has already been mentioned, the swelling behavior in water of the samples would affect the following drug loading process. According to these data, CPN swelled to a high extent in water and logically exhibited a high DSP loading capacity.

It must also be noted that drug loading was achieved by swelling of the networks in DSP aqueous solutions of varied concentrations (2.5% and 5.0%), with the objective being to determine whether different concentrations of the aqueous DSP solution would affect the DSP content in the networks. As can be seen from Figure 4, doubling the concentration of aqueous DSP solution resulted in doubling the DSP content (%) in the networks. This could be considered as an exceptional advantage of the synthesized networks, because it would allow, by varying the concentration of the drug aqueous solution, different loading efficiencies to be achieved depending on the needs.

**Figure 5:**
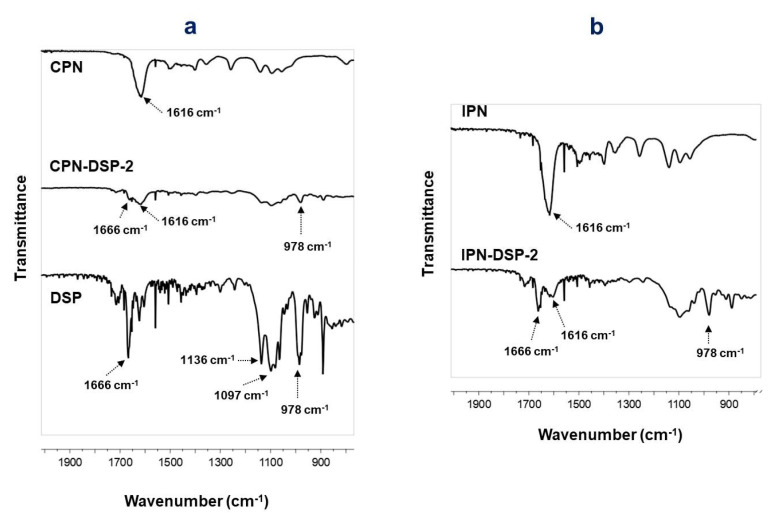
FTIR spectrum of DSP-loaded CPN compared to the spectra of neat CPN and pure DSP (**a**), and FTIR spectrum of DSP-loaded IPN compared to the spectrum of neat IPN (**b**).

FTIR spectra of loaded CPN and IPN were analyzed in order to investigate possible drug–polymer interactions. As it can be seen from the spectra presented in Figure 5, no interactions between the drug and the polymers were observed, as there were no shifts in the characteristic bands of the networks and DSP.

### 2.5. Loading Efficiency

The loading efficiency (LE, %) was calculated based on the results obtained for the DSP content in the networks. There was a difference in the LE of the individual networks, which is logical given the difference in swelling kinetics and DSP content. The highest LE was observed for CPN-DSP-2, loaded by swelling in 5% DSP aqueous solution—49.36%, and for the IPN-DSP-2 it was 34.29%, respectively. In the case of networks loaded by swelling in 2.5% aqueous DSP solution, the CPN-DSP-1 was characterized by higher LE—23.33%, compared to the IPN-DSP-1 with 17.95%.

### 2.6. Thermogravimetric Analysis (TGA)

**Figure 6:**
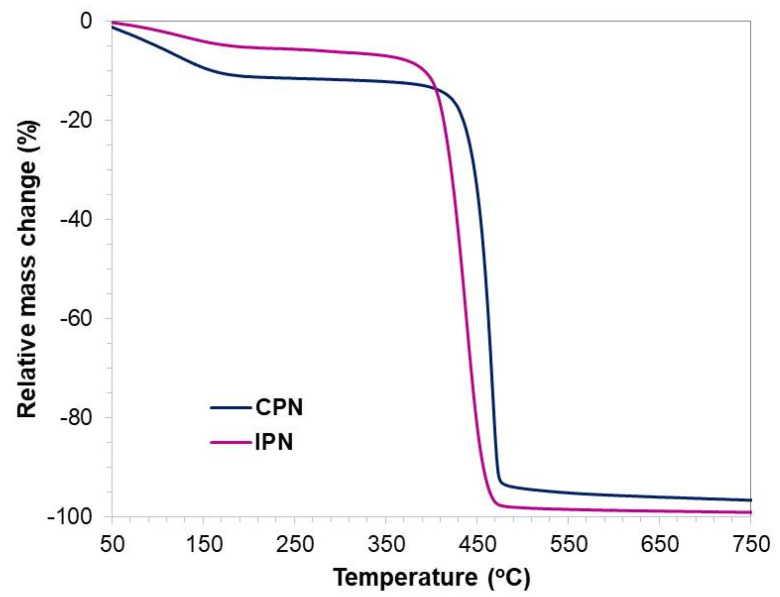
TGA curves of pure CPN and IPN.

The bulk thermal stability and compositional properties of the networks were assessed using thermogravimetric analysis. The thermal degradation profiles of CPN and IPN are presented in Figure 6. The initial weight loss (about 10% for CPN and 5% for IPN) in the interval from 50 °C to 270 °C was attributed to the elimination of solvent molecules and volatile matters from the complex network structure.

As can be seen in Figure 6, both networks were thermally stable up to 400 °C, as a result of the successful crosslink polymerization reactions during preparation of the networks. CPN’s main weight loss (83%) appeared in a narrow temperature interval from 430 °C to 470 °C, and may be attributed to the decomposition of the PDMAAM and PEO functional groups (amide and ether) and backbones, as well as to the network de-crosslinking. For IPN, degradation starts at a lower temperature (at about 400 °C) and resulted in 93% mass loss in a similar narrow temperature interval. Although CPN and IPN were built of segments of different chemical structures and molar mass characteristics, decomposition occurred homogeneously in one step. This indicated that different building blocks in the network structure were bonded and compatibilized at a macromolecular level.

**Figure 7:**
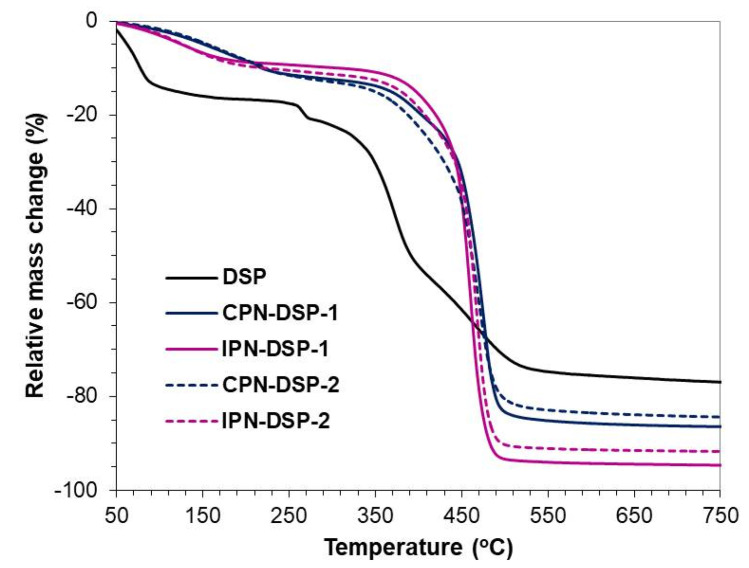
TGA of pure DSP and DSP-loaded CPN and IPN.

Thermal degradation profiles of pure DSP and DSP-loaded CPN and IPN are presented in Figure 7. The TGA curve of pure DSP clearly shows three stages of weight loss: the first, in the interval from 50 °C to 250 °C, could be attributed to the evaporation of adsorbed water [53]. The second one—from 250 °C to 450 °C—and the third one, from 400 °C to 500 °C, were obviously due to the decomposition occurring in different functional groups of the DPS molecule. A char residue of 25.8% was estimated at the end of the DSP thermal analysis. When intercalated in the studied networks, DPS molecules demonstrated enhanced thermal stability, as can be concluded from the degradation profiles of CPN-DSP-1, CPN-DSP-2, IPN-DSP-1, and IPN-DSP-2 (Figure 7). Although containing a considerable amount of DSP (from 17 to 50 wt.%), drug-loaded networks showed single-stage decomposition in the interval from 400 °C to 470 °C, similar to pure CPN and IPN (Figure 6 and Figure 7). The char residue was negligible in the case of pure CPN and IPN (Figure 6); however, in the drug-loaded networks it increased proportional to the amount of intercalated DSP content (Figure 7).

### 2.7. In Vitro Drug Release Study

**Figure 8:**
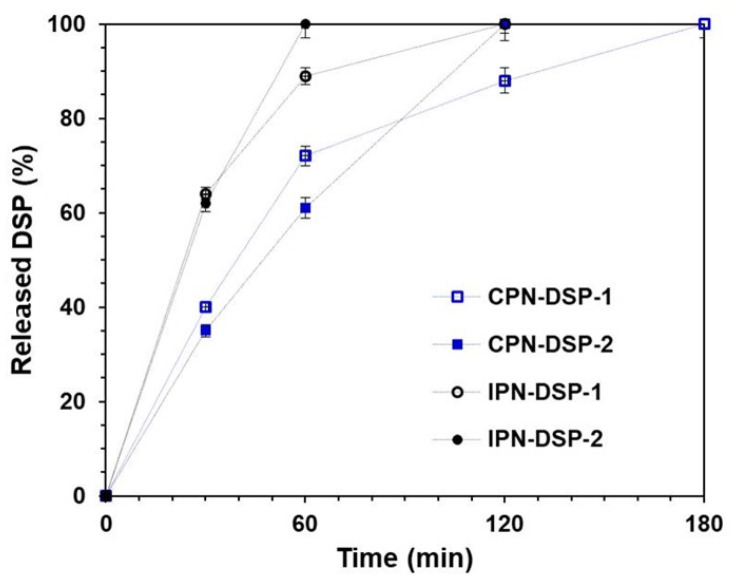
DSP release from CPN and IPN at 37 °C in BS with pH 7.4.

Drug release kinetics of DPS loaded networks studied in vitro at 37 °C in BS with pH 7.4 are presented in Figure 8. A significant burst-effect was observed for both networks. It is obvious that in the first hour, the amount of DSP released was different. A higher release rate from IPN was established, compared to CPN. The reason for the differences in the rate and degree of drug release from CPN and IPN can be explained based on the results obtained from the swelling kinetics. As has already been noted, in the medium in which the drug release study was performed (BS at 37 °C) the temperature-responsive PNIPAAM segments of the second network in IPN were collapsed and provided additional volume in the IPN pore structure for the BS solution, through which the loaded DSP could be released faster.

It must also be pointed out that the investigated networks released up to 100% of the loaded DSP within 3 h. A greater prolongation in the DSP release rate was observed for CPN (especially with CPN-DSP- 2 with a higher DSP content), and the burst effect in this case was significantly reduced compared to the other networks. This means that in cases where the situation required a faster effect, IPNs were more suitable. Conversely, if a longer and more uniform release of the drug substance is required, CPN is more suitable. Therefore, in order to precisely control the drug release, it is necessary to improve the characteristics of CPN.

### 2.8. Drug Release Kinetics

The mathematical modelling of drug delivery is a tool used to understand the release behavior of different pharmaceutical delivery systems. In order to determine the drug release profiles, different kinetic models such as Zero-order, First-order, Higuchi, and Korsmeyer–Peppas were used in this study.

The fitting of in vitro release data into different kinetic models was used to establish the value of the correlation coefficient (R^2^). Based on this value, the different models were compared as a value closer to 1 indicated a better correlation. In Table 1 are listed the correlation coefficients (R^2^), the constants of the different models, and the release exponent (*n*) obtained after fitting the experimental data of DSP release from the different networks.

For all studied types of networks, it was established that the DSP release was best characterized by the Higuchi model (R^2^ > 0.95). According to this model, the decline in the drug release over time was due to the formation of a layer upon the surface of the drug. The data showed that DSP release from the polymer networks was a diffusion-controlled process. Furthermore, high values of *k_H_* are associated with a substantial release rate at the beginning of the release process. From the results presented in Table 1, it can be seen that the values of *k_H_* for all polymer networks were relatively high (above 15), which was an indication for a significant burst release. This was also confirmed by the drug release experiments.

Since it was established that the prime mechanism of drug release was diffusion-controlled, the type of diffusion must be specified. Therefore, the release data were fitted using the equation derived by Korsmeyer and Peppas. According to this model, the constant *k_KP_* varies depending on the characteristics of the system, and the type of diffusion could be determined by the value of the release exponent (*n*). In the case when *n* ≤ 0.5, the release is prevailed by the Fickian diffusion mechanism; in the case when 0.5 ≤ *n* ≤ 1, the release is characterized by an abnormal diffusion (non-Fickian diffusion), and when *n* > 1, the release follows a complex transport mechanism (super-case-II transport). The results presented in Table 1 indicated, that the release followed a complex transport mechanism (super-case-II transport) (*n* > 1).

## 3. Conclusions

In the present work, CPN and IPN were obtained by combining free radical polymerization and redox polymerization. The chemical composition of the prepared networks was confirmed using FTIR spectroscopy. The bulk thermal stability of the networks was assessed with TGA. The swelling kinetics of the copolymer networks were investigated in water, in BS (pH 7.4) and in ethanol. The data from the swelling experiments in water revealed that the investigated platforms could absorb considerable amounts of water. CPN showed lower equilibrium swelling in BS, but followed an identical kinetic profile to those in water, while the swelling of IPN in BS at 37 °C resulted in equilibrium swelling almost equal to the swelling in pure water. Both CPN and IPN swelled equally in ethanol regardless of the copolymer composition, but much lower compared to their swelling in water and BS. The prepared networks were drug loaded via swelling in DSP aqueous solution. IPN showed a lower loading efficiency compared to CPN. The data from the in vitro drug release studies showed a significant burst-effect for both networks. A higher release rate from IPN was established, compared to CPN. It must also be pointed out that the studied networks released up to 100% of the loaded DSP within 3 h. The conducted drug release kinetics study established that DSP release from the samples was best described by the Higuchi model (R^2^ > 0.95). According to the Korsmeyer and Peppas model, the release followed a complex transport mechanism (super-case-II transport). The results from the conducted study indicate that the prepared copolymer networks have potential for DSP delivery, and will be subjected to further in vivo investigation for dermal applications.

## 4. Materials and Methods

### 4.1. Materials

Monomers N,N-dimethylacrylamide (DMAAM), N-isopropylacrylamide (NIPAAM), and poly(ethylene glycol) (PEG; average molar mass 1900–2200), crosslinker poly(ethylene glycol) diacrylate (PEGDA; average Mn 600), and initiator 4,4′-azobis(4-cyanovaleric acid) (≥98%) were purchased from Sigma-Aldrich (Seelze, Germany). Initiator cerium(IV) ammonium nitrate (CAN) was obtained from Fisher Chemical (Pittsburgh, PA, USA). Other solvents and reagents used were of standard analytical reagent grade. Dexamethasone sodium phosphate was purchased from Crystal Pharma (Valladolid, Spain).

### 4.2. Methods

#### 4.2.1. Synthesis of Multicomponent Polymer Networks

First, copolymer network (CPN) was synthesized by redox polymerization of N,N-dimethyl acrylamide (DMAAM) initiated by CAN onto poly(ethylene glycol) (PEG) as di-functional hydroxyl containing precursor and in the presence of poly(ethylene glycol) diacrylate (PEGDA) as crosslinker. Prior to the polymerization, the monomer DMAAM was passed through an alumina column for the removal of inhibitor traces. A reaction mixture containing DMAAM, PEG, and PEGDA dissolved in water (initial DMAAM concentration 5 g/L; mole ratio DMAAM:PEG:EGDA = 70:1:0.2) was prepared and purged with nitrogen for 15 min for oxygen elimination. A solution of the initiator CAN (in equimolar amount to the PEG hydroxyl groups) in 1N HNO_3_ was prepared and added to the reaction mixture under nitrogen. Nitrogen bubbling was continued for another 15 min and polymerization was implemented in the flask for 1 h at 35 °C under nitrogen atmosphere. Then, the reaction mixture was transferred to the film mold and polymerization was allowed to continue at 35 °C for 24 h. The gel was removed from the reaction mold and dried in vacuum. Raw films were washed of reaction residues by soaking in deionized water for a week, water being replaced every 3 h. The obtained purified copolymer network films were dried in vacuum until constant weight.

For the preparation of IPN, a reaction mixture of NIPAAM and PEGDA in deionized water was prepared (initial NIPAAM concentration 5 g/L; mole ratio NIPAAM:EGDA = 70:0.2) and bubbled with nitrogen for 15 min. A solution of water-soluble radical initiator 4,4′-azobis(4-cyanovaleric acid) was added, and samples of previously synthesized CPN were allowed to swell in this reaction mixture for 24 h under nitrogen at 10 °C. The procedures for the synthesis of CPN were then followed and obtained purified IPN samples were dried in vacuum until constant weight.

#### 4.2.2. Fourier Transform Infrared Spectroscopy (FTIR)

Fourier transform infrared (FTIR) spectra were recorded on an IRAffinity-1 spectrophotometer (Shimadzu Company, Kyoto, Japan). The equipment is supplied with a MIRacleTM ATR accessory (diamond crystal; PIKE Technologies, Madison, WI, USA) providing a depth of penetration of the IR beam into the sample of about 2 μm. Network sample films were analyzed in the wavenumber range from 4000 cm^−1^ to 600 cm^−1^, performing 50 scans at a resolution of 4 cm^−1^.

#### 4.2.3. Swelling Experiments

Swelling kinetics of the developed multicomponent networks were investigated in water, ethanol, and in buffer solution (pH 7.4). Swelling degrees were estimated by performing at minimum 3 parallel measurements of each network. Swelling degree *Q* (%) was calculated using the following equation, where *w*_0_ and *w* are the weight of the dry and swollen sample, respectively:

(1) $$Q\left(\%\right)=100\;\frac{w-w_{0}}{w_{0}}\;$$

Soluble fractions (*SF*) were calculated using the following equation, where *w*_0_ and *w_d_* are the weight of the initial dry sample and sample dried after swelling experiment, respectively:

(2) $$SF\left(\%\right)=100\;\frac{w_{d}-w_{0}}{w_{0}}$$

#### 4.2.4. Drug Loading and DSP Content

Networks were drug-loaded by swelling in DSP aqueous solutions of varied concentrations (2.5% and 5.0%). The networks were left for 24 h in DSP aqueous solution with different concentrations. After reaching the equilibrium degree of swelling, the samples were taken out, weighed on an analytical balance, and then dried under vacuum for 24 h to a constant weight. The DSP content was calculated using the initial weight of the dry network and the weight of the dried sample.

#### 4.2.5. Loading Efficiency (%)

The loading efficiency (*LE*, %) was calculated based on the results obtained for the DSP content using the following equation:

(3) $$LE\;\left(\%\right)=100\frac{weight\;DSP\;loaded}{initial\;weight\;DSP}\;$$

#### 4.2.6. Thermogravimetric Analysis (TGA)

Thermal properties of bulk networks were investigated using a TGA-4000 Perkin Elmer Thermogravimetric Analyser (PerkinElmer, Inc., Waltham, MA, USA). Film samples ranging from 12 to 14 mg were examined from 40 °C to 800 °C under nitrogen flow at a heating rate of 10 °C/min.

#### 4.2.7. In Vitro Drug Release Study

Drug release experiment was conducted in a water shaking bath (IKASH-B20, Staufen, Germany) at a rotation speed of 50 rpm, and maintained at 37 ± 0.5 °C, in 100 mL BS (pH 7.4). Samples (2 mL) were taken at predetermined time intervals until 100% DSP release was established. The quantity of DSP was determined with UV spectroscopy (absorbance at 240 ± 2 nm) using a Hewlett-Packard 8452 A diode array spectrophotometer (Palo Alto, CA, USA). The percentage of drug released was calculated using the data obtained from the studies of each network, conducted in triplicate.

#### 4.2.8. Drug Release Kinetics

In order to establish the drug release profiles, four kinetic models, Zero-order, First-order, Higuchi, and Korsmeyer–Peppas, were used.

- Zero-order model—this model is applied on occasions when the drug release rate is not dependent on its concentration.

(4) $$\frac{M\left(t\right)}{M\left(\infty \right)}=k_{0}t\;$$

- First-order model—according to this model, the drug release rate is directly proportional to the drug concentration. The first-order process is characterized by linear kinetics.

(5) $$\frac{M\left(t\right)}{M\left(\infty \right)}=e^{-k_{1\;}t}\;$$

- Higuchi model—Higuchi suggested that two mechanisms are responsible for controlling the drug release rate: swelling and erosion/degradation.

(6) $$\frac{M\left(t\right)}{M\left(\infty \right)}=k_{H}t^{\frac{1}{2}}$$

- Korsmeyer–Peppas model—this model is used to describe the process when the release follows several kinetics mechanisms.

(7) $$\frac{M\left(t\right)}{M\left(\infty \right)}=k_{KP}t^{n}$$

In Equations (4)–(7), *M*(*t*) represents the amount of DSP released at time *t* and *M*(*∞*) represents the total amount of DSP loaded in the networks; *k*_0_, *k*_1_, *k_H_*, and *k_KP_* are the constants of Zero-order, First-order, Higuchi, and Korsmeyer–Peppas models, respectively.

## Figures and Tables

**Table 1:** Parameters describing the drug release kinetics.

Kinetic Model	CPN-DSP-1	CPN-DSP-2	IPN-DSP-1	IPN-DSP-2
Zero-order	*k*_0_ = 3.9223 R^2^ = 0.8368	*k*_0_ = 4.2645 R^2^ = 0.8584	*k*_0_ = 2.3058 R^2^ = 0.6393	*k*_0_ = 7.2929 R^2^ = 0.9815
First-order	*k*_1_ = 2.6353 R^2^ = 0.7764	*k*_1_ = 3.3339 R^2^ = 0.9175	*k*_1_ = 2.0223 R^2^ = 0.8035	*k*_1_ = 1.2990 R^2^ = 0.4576
Higuchi	*k_H_* = 18.1229 R^2^ = 0.9692	*k_H_* = 15.3779 R^2^ = 0.9971	*k_H_* = 23.0216 R^2^ = 0.9513	*k_H_* = 20.0597 R^2^ = 1
Korsmeyer–Peppas	*n* = 1.1519 R^2^ = 0.8507	*n* = 1.3031 R^2^ = 0.9898	*n* = 2.8795 R^2^ = 0.9305	*n* = 1.4469 R^2^ = 1

## Data Availability

Not applicable.
